# Co-designing a Self-Management App Prototype to Support People With Spinal Cord Injury in the Prevention of Pressure Injuries: Mixed Methods Study

**DOI:** 10.2196/18018

**Published:** 2020-07-09

**Authors:** Julia Amann, Maddalena Fiordelli, Mirjam Brach, Sue Bertschy, Anke Scheel-Sailer, Sara Rubinelli

**Affiliations:** 1 Swiss Paraplegic Research Nottwil Switzerland; 2 Health Ethics and Policy Lab Department of Health Sciences and Technology ETH Zurich Zurich Switzerland; 3 Institute of Communication and Health University of Lugano Lugano Switzerland; 4 Department of Health Sciences and Medicine University of Lucerne and Swiss Paraplegic Research Lucerne Switzerland; 5 Swiss Paraplegic Center Nottwil Switzerland

**Keywords:** co-design, mHealth, eHealth, self-management, spinal cord injury, pressure injury

## Abstract

**Background:**

Spinal cord injury is a complex chronic health condition that requires individuals to actively self-manage. Therefore, an evidence-based, self-management app would be of value to support individuals with spinal cord injury in the prevention of pressure injuries.

**Objective:**

The main objectives of this study were to (1) establish a co-design approach for developing a high-fidelity prototype app for the self-management of individuals with spinal cord injury, (2) design the prototype that resulted from this process, and (3) conduct the first usability assessment of the prototype app.

**Methods:**

We adopted a co-design approach to develop an evidence-based app prototype. Starting from a preliminary content model (based on clinical guidelines for the prevention of pressure injuries) and three research-based user personas, we conducted an ideation workshop involving individuals with spinal cord injury and health care professionals. The ideation workshop formed the basis for two consecutive design sprints. The result of this co-design phase was an interactive app prototype. The prototype was evaluated in two rounds of usability testing (N=4 and N=15, respectively) using a combination of qualitative and quantitative methods.

**Results:**

The co-design process resulted in a high-fidelity prototype with two key components: a self-management component and a communication component. The final prototype included a combination of features to support individuals with spinal cord injury in the prevention of pressure injuries, namely a smart camera, pressure injury diary, expert consultation, reminders, and knowledge repository. Findings of the usability testing showed that most participants navigated the app fluently with little back and forth navigation and were able to successfully complete a set of assigned tasks. These positive results are supported by the average system usability score achieved (78.5/100; range 47.5-95.0) and our qualitative analysis of the semistructured interviews. Despite an overall positive evaluation of the app prototype, we identified areas for improvement (eg, inclusion of a search function).

**Conclusions:**

Individuals with spinal cord injury often need to navigate competing interests and priorities, paired with uncertainty about the accuracy and relevance of clinical recommendations. Understanding what matters to individuals with spinal cord injury can help guide the design of behavioral interventions that are useful and acceptable to these individuals in their daily lives. This study shows that involving individuals with spinal cord injury and health care professionals in co-designing a self-management app can foster knowledge cocreation at the intersection of lived experience, medical expertise, and technical solutions.

## Introduction

Spinal cord injury is a complex chronic health condition that also makes individuals prone to several secondary complications, including pressure injuries [1-3]. In fact, pressure injuries are one of the most common secondary complications affecting community-dwelling individuals with spinal cord injury [3-5]. In addition to having a considerable impact on a person’s health, quality of life, and well-being, there are also high economic costs associated with the treatment of pressure injuries [4,6]. Several risk factors associated with pressure injuries have been recognized, including sociodemographic, neurological, functional, clinical, biological, and medical care management [7]. Some of these factors such as age or lesion level are nonmodifiable. For example, natural skin aging increases the risk of developing a pressure injury.

In this context, some researchers have highlighted the key role that self-management plays in the prevention of pressure injuries [2,8]. Indeed, several guidelines and recommendations exist to guide individuals on how to prevent pressure injuries [9]. Yet, research indicates that individuals with spinal cord injury may not always follow evidence-based recommendations regarding physical activity, diet, and other preventive measures (eg, regular skin checks and pressure relief) once they return to their daily routine after the first rehabilitation [10-12]. These findings may be explained by considering that individuals with spinal cord injury adopt different prevention styles, characterized by different preventive behaviors and attitudes toward pressure injuries and prevention more generally [13]. Understanding what matters to individuals is essential to ensure that interventions are acceptable and rooted in the reality of the individuals’ daily lives [14]. In light of this, it has become evident that self-management support needs to be tailored to individual prevention styles to motivate and engage individuals.

Given that self-management programs have long focused on equipping individuals with the knowledge and skills they need to manage chronic health conditions rather than seeking to understand how to best accommodate different life situations, it comes as no surprise that there is no conclusive evidence on how to best support community-dwelling individuals with spinal cord injury in the prevention of pressure injuries [15,16]. Self-management can be broadly defined as “the tasks that individuals must undertake to live with one or more chronic conditions. These tasks include having the confidence to deal with medical management, role management and emotional management of their conditions” [17]. In this paper, we consider self-management as a set of activities and behaviors that an individual actively performs or avoids so as to prevent or alleviate symptoms related to pressure injuries.

In recognizing the complexity and fragmentation of evidence-based and preference-sensitive information that is relevant for a person to effectively self-manage, it becomes clear that a conventional top-down approach to self-management support may no longer be adequate. Rather, there is a need to engage different stakeholder groups in the development of self-management programs to ensure that solutions meet the requirements for successful implementation and favorable health outcomes [14]. Accordingly, there has been a significant increase in participatory approaches to design and improve self-management programs for individuals with chronic health conditions [18-21]. A particular area of research that has developed from this trend for participatory approaches is the co-design of mobile health (mHealth) apps [22-25]. Several recently published studies present different approaches to co-designing self-management apps for health conditions, including asthma [26], cystic fibrosis [27], sickle cell disease [23], and spinal cord injury [28]. Arguably, co-design has intuitive appeal and is promoted on a political level. The normative assumption is that the outcome of solutions that are co-designed by users and professionals ought to be better [29]. Yet, there is limited research on how to effectively engage medically fragile populations in generating user specifications for mHealth tools to foster self-management [30,31].

Guided by this literature, we aimed to develop an evidence-based, self-management app to support individuals with spinal cord injury in the prevention of pressure injuries. In this paper, we describe the co-design approach used, present the app prototype that resulted from this process, and report the findings of a first usability assessment. We conclude by critically reflecting on co-design as an approach to enrich the development of self-management apps for individuals with disabilities more generally. This study constitutes part of a larger project on the prevention of pressure injuries in community-dwelling individuals with spinal cord injury in Switzerland.

## Methods

### Study Design

We followed a co-design and development approach similar to that described by Gray et al [30], incorporating qualitative research methods (ie, semistructured interviews) into user-centered design approaches (ie, ideation workshop, design sprints, usability tests). The study was approved by the Cantonal Ethics Commission (EKNZ 2017-01787).

**Figure 1:**
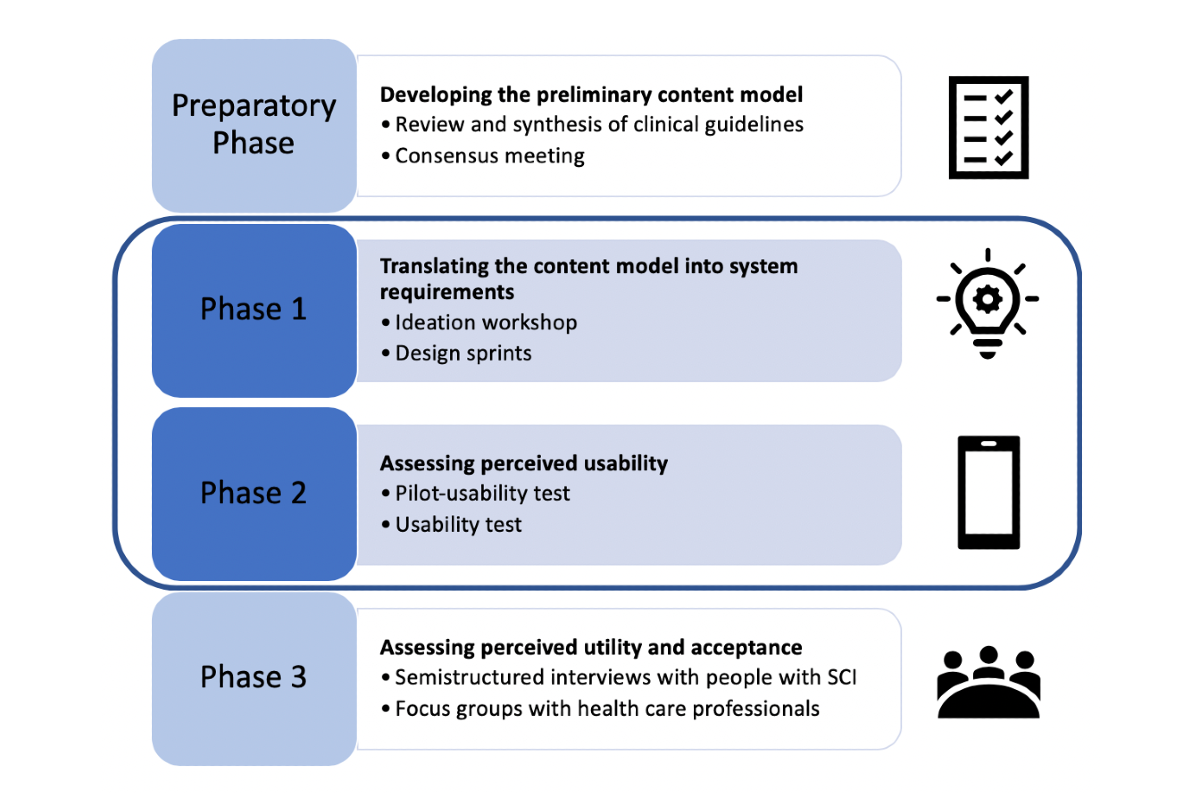
Co-design and development approach. SCI: spinal cord injury.

In a preparatory phase, we involved different stakeholders in assessing available clinical guidelines for the prevention of pressure injuries in people with spinal cord injury. Based on the findings of this preparatory phase, we developed a preliminary content model for the app prototype. In Phase 1, this preliminary content model was translated into concrete functional and nonfunctional requirements of the app prototype. In Phase 2, we performed a usability test to assess the perceived usability of the app prototype. In Phase 3, we conducted semistructured interviews and focus groups with health care professionals to assess the utility of the app prototype and to determine whether it could be implemented in practice. An overview of our methodological approach is displayed in Figure 1. The present paper describes the outcomes of Phase 1 and Phase 2. Methods and findings of the preparatory phase have been published elsewhere [9].

### Preparatory Phase: Developing a Preliminary Content Model

To identify the most relevant guidelines for the prevention of pressure injuries that community-dwelling individuals with spinal cord injury can and should perform, we held a consensus meeting involving 15 health care professionals specialized in spinal cord injury. In addition, two individuals with spinal cord injury and an insurance representative participated in the meeting. The consensus meeting resulted in a set of 98 guidelines spread across 12 categories: (A) Support surface, (B) Repositioning, (C) Nutrition, (D) Skincare, (E) Skin assessment, (F) Exercising, (G) Collaboration with health professionals/caregivers, (H) Transfers, (I) Clothing, (J) Body function and structure, (K) Personal factors, and (L) General. This selection of clinical guidelines formed the preliminary content model of the app (for detailed results see [9]). In other words, these guidelines served as building blocks that would guide the development of the evidence-based app content.

### Participants (Phase 1 and Phase 2)

Through the different phases of the project, we aimed for balanced participant samples in terms of age (>18 years), gender, and lesion level, and health care professionals’ expertise. Individuals with spinal cord injury and health care professionals were recruited with the support of health professionals from the Swiss Paraplegic Center (in particular members of the interdisciplinary Decubitus-Care team), participants of the consensus meeting described above, as well as through support of the Swiss Paraplegic Association and Parahelp, a home care service provider specialized in spinal cord injury care. This recruitment strategy was complemented by an online call for participants published through the Paraplegie Community [32], an online community for individuals with spinal cord injury. People who expressed interest to participate in the study were contacted by email or telephone. All participants received detailed study information and were asked to sign a consent form. We did not offer any financial incentives. Table 1 presents information on the study populations of Phase 1 and Phase 2.

**Table 1:** Study participants in Phase 1 and Phase 2.

Phase		N
**Ideation workshop (Phase 1)**		
	Project team	2
	Parahelp home care provider	2
	Wound specialist	1
	Nutritionist	1
	People with SCI^a^	5
	User experience designer	2
**Design sprints (Phase 1)**		
	Project team	4
	User experience designer	2
**Usability test (Phase 2)**		
	Pilot test	4
	Usability test	15

^a^SCI: spinal cord injury.

### Phase 1: Translating the Content Model Into System Requirements

The key challenge was then to translate the content model into concrete functional and nonfunctional requirements of an app prototype that would be perceived as useful and acceptable by prospective users. To this end, we collaborated closely with individuals with spinal cord injury, health care professionals specialized in spinal cord injury, as well as user experience designers. The translation process consisted of two main activities.

First, we held a 1-day ideation workshop with health care professionals, researchers, people with spinal cord injury, and user experience designers. The workshop pursued two specific aims: (1) to translate the preliminary content model into concrete functions, and (2) to foster stakeholder engagement and commitment to the project. For this purpose, we sought to gain an in-depth understanding of the challenges faced by the different stakeholders in the prevention of pressure injuries and to collect ideas on how an app could address these challenges.

The ideation workshop consisted of a set of individual and group activities commonly used in user experience design [33] and was moderated by a trained user experience designer. We aimed to encourage participants to draw on their own experiences and expertise but also to reflect on those of the other participants. Activities incorporated the evidence-based content model developed in the preparatory phase [9] and 3 research-based user personas [34,35] to stimulate discussion. User personas are fictitious characters to represent different prospective user types who are characterized by specific goals and behaviors [35]. The user personas adopted for this study described the three different prevention types that had been identified in earlier work: the thoughtful, the selective, and the delegator [13]. Each prevention type is characterized by different preventive behaviors, knowledge, and attitudes toward the prevention of pressure injuries; collaboration with health care professionals; and attitudes toward spinal cord injury in general [13]. For example, in one of the exercises, participants worked in smaller groups to create a list of app features and functionalities that would be perceived as “cool” or “not cool” by the respective type their group had been assigned. In another activity, participants were again divided into smaller groups to brainstorm how specific guidelines might be translated into concrete contents and functions (ie, identify opportunities and challenges). As individual activities, participants had to write a “love letter” and a “one-star review,” respectively, to describe what they liked and disliked about the (at this point) fictional app. Moreover, we asked participants to sketch their ideas for user interfaces to activate their creative thinking and problem-solving skills. Impressions from the ideation workshop are presented in Multimedia Appendix 1.

Following the ideation workshop, we conducted two consecutive design sprints [36]. During these design sprints, the research team and user experience designers aimed to synthesize, condense, and prioritize the ideas that were generated during the ideation workshop. For this purpose, we reviewed the material collected during the ideation workshop individually and then as a group. We revisited the user journey and potential use cases. Finally, we used a feasibility-impact matrix [37] to guide priority setting in selecting the functions to be implemented in the first iteration of the app prototype. More specifically, we mapped the different features and functionalities (written on Post-It notes) onto a physical easy/hard–low/high impact matrix to determine those that were high impact (ie, with great potential benefit for the user) and feasible (ie, easy to implement). Features that were deemed high impact but difficult to implement for technical reasons (eg, integrated ruler in the smart camera to indicate the size of the pressure injury) were not included in the first iteration of the app prototype. Following this process, the first prototype in the form of a clickable user interface was developed.

Data that were collected during the ideation workshop and design sprints included: (1) written Post-It notes, (2) participants’ drawings, and (3) field notes taken by the research team. We used an online project management tool (RealTime Board) to collaboratively collate, synthesize, and analyze the materials in a comparative process akin to thematic analysis [38], together with user experience designers. In our analysis, we focused on capturing participants’ needs and desires, as well as ideas for concrete functions and features. We then followed a narrative approach to group similar ideas and concepts into overarching, inductively derived categories that would ultimately form the structural model of the app prototype.

### Phase 2: Assessing Perceived Usability

The central objective of Phase 2 was to examine the perceived usability and usefulness of the app from the perspective of potential service users. In this phase, we also aimed to collect suggestions for improvement and ideas for designing the functions, content, and navigation of the app. A pilot usability test was carried out with 4 individuals with spinal cord injury using a first iteration of the app prototype. Based on the findings of the pilot test, a second and more refined version of the prototype was developed and then tested by 15 individuals with spinal cord injury. Table 2 presents an overview of the participants’ characteristics.

**Table 2:** Characteristics of study participants in Phase 2.

Characteristic		Pilot test (N=4)	Usability test (N=15)
Age (years), mean (range)		55.5 (48-64)	40.8 (28-58)
**Gender, n (%)**			
	Male	3 (75)	11 (73)
	Female	1 (25)	4 (27)
**Lesion level, n (%)**			
	Paraplegic	2 (50)	7 (47)
	Quadriplegic	2 (50)	8 (53)

Both usability tests were carried out in a laboratory setting (with one exception that was carried out at the person’s home) following the same procedure (Textbox 1), which was informed by earlier work on mHealth usability studies [39]. In particular, we combined two common usability-testing approaches, task completion [40] and think-aloud technique [41,42], with additional observational data on navigation fluidity and navigation challenges. In doing so, we aimed to determine whether the app prototype was designed in such a way that users were able to fluently navigate it. We complemented these assessments with a semistructured interview and a self-administered questionnaire to gain further insights into how participants perceived the usability of the app prototype. Upon completion of the pilot test, the app prototype was also presented to the individuals with spinal cord injury and health care professionals that had taken part in the ideation workshop to share with them the outcome and to collect informal feedback.

**Procedure for usability testing.**

**Usability Test**

As part of the usability test, participants were asked to complete a series of 11 tasks using the app prototype. The 11 tasks used were chosen as they represent typical use cases (eg, “Set a reminder for a mattress check for January 30, 2019”). While completing the tasks, participants were instructed to verbalize their navigation behavior (“think-aloud technique”) [43] (eg, where they look for certain information or where they would expect to find it). Participants with limited hand functioning were provided with an assistive pen to facilitate navigation. A structured observation sheet was developed by the research team through internal pretesting and expert recommendation provided by the user experience designer. Prior to data collection, a research assistant received detailed coding instructions. During the usability test, the following data were collected: (1) task completion (ie, did the participant successfully complete the task, rated as concluded, partially concluded, not concluded); (2) task completion time (ie, time required to complete the task successfully, recorded as time in seconds); (3) navigation fluidity (ie, how fluently participants were navigating the app prototype, rated on a 5-point scale from “irregular”=1 to “fluent”=5); and (4) challenges related to completion of the task (observed and stated by participants, and noted down by a research assistant).

In addition, participants’ navigation behavior and commentary were recorded using a screen camera, screen-capturing software, and audio-recording device (see Multimedia Appendix 2). After data collection was complete, a second coder (JA) went back to the coding to ensure intercoder reliability.

**Semistructured interview**

Following the usability test, participants took part in a semistructured interview guided by the technology acceptance model [44], which aimed to capture participants’ views regarding usability and perceived usefulness of the app prototype, as well as their attitudes and intentions toward using the app (see Multimedia Appendix 3). Moreover, we aimed to elicit ideas and suggestions for improvement. All interviews were transcribed verbatim and analyzed using inductive thematic analysis [38].

**Questionnaire**

Finally, the study participants were asked to complete a short questionnaire on system usability. The questionnaire consisted of 10 items to be rated on the system usability scale (SUS), which is a 10-point Likert scale [26]. The SUS score was calculated for each participant.

## Results

### Phase 1: Content and Functionalities of the App Prototype

Within the scope of the co-design process, we identified two key components of the app. The first is a self-management component that would need to support individuals in documenting their pressure injuries, including visual and written information (disease monitoring); finding relevant, evidence-based information on pressure injury prevention and related topics (disease knowledge); and receiving personalized and actionable recommendations based on their preferences and needs (motivational support). The second is a communication component that would allow users to safely and quickly get in touch with a health care provider to inquire about pressure injury-related topics (eg, nutrition) and send pictures and information on pressure injuries to receive feedback.

In the subsections below, we describe the key functionalities of the app prototype that we identified as necessary to achieve the goals of these two components. An overview is provided in Multimedia Appendix 4. Note that the prototype presented here is the iteration used for the usability test in Phase 2.

#### Smart Camera

**Figure 2:**
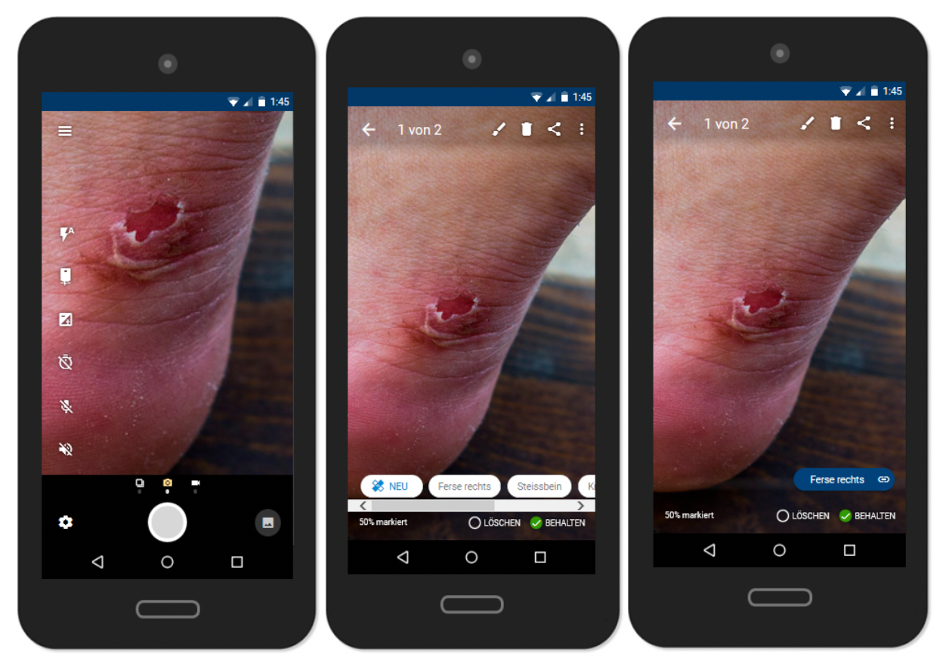
Smart camera.

A central function of the app is the smart camera (Figure 2). Pictures and videos taken with the smart camera can be directly added to the pressure injury diary, the documentation function of the app, or forwarded to a health care provider.

The need for a smart camera resulted from a vivid discussion around the poor quality of pictures often taken by people with spinal cord injuries. Particularly, health care professionals emphasized that they need high-quality pictures to be able to make reasonable judgments about the condition and state of a pressure injury when providing advice remotely. Based on these discussions, several additional features envisioned by the workshop participants emerged, including a voice-operated shutter release function, which would be particularly useful for people with limited hand functioning; a multi-shot mode; and tips and tricks for taking good pictures (eg, paying attention to light conditions, angles, and image sharpness). Despite the desire of participants to have an integrated ruler to indicate the size of the pressure injury, this function could not be implemented due to technical constraints. It was thus suggested to instead provide recommendations on how size can be inferred, for example by placing a coin next to the pressure injury or by using self-adhesive ruler tape.

Even though health care professionals considered a function that would help users take better pictures as highly valuable, they also emphasized that pictures cannot replace a physical exam but rather provide some indication of the urgency of the situation. This was also a warning message they would like to see integrated into the final smart camera. An additional camera feature that people with spinal cord injury desired for privacy reasons was the “censoring function,” which is a tool that allows users to easily edit pictures using a black marker, which might be desired to hide intimate body areas as an example. The individuals with spinal cord injury also emphasized that sometimes they may just want to check their skin using their smartphone camera as a mirror, rather than saving or sharing pictures. This is why pictures and videos are not saved automatically into the pressure injury diary.

#### Pressure Injury Diary

**Figure 3:**
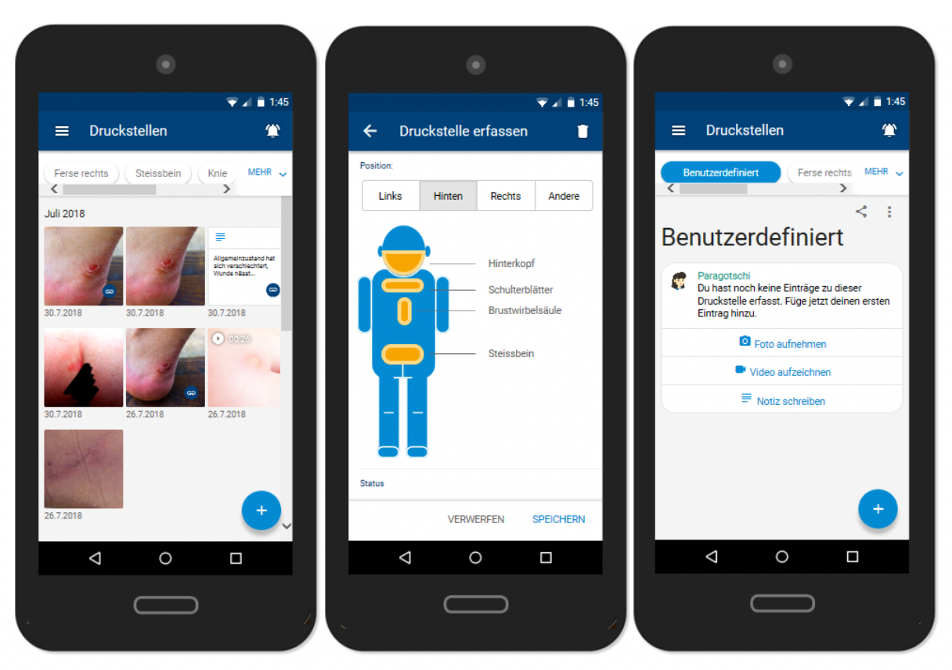
Pressure injury diary.

The pressure injury diary, as the documentation function of the app, allows users to store and manage their pictures, videos, and other relevant information such as date, time, and location of the pressure injury. The documentation function is intended to support users in the early detection and observation of conspicuous skin areas. New entries can be entered directly using the camera function (as shown in Figure 2) or in the “My pressure injuries” section (Figure 3). In addition to pictures, personal notes can be added. When adding a picture, participants are also prompted to add additional information, including the location of the pressure injury and the presumed cause of the injury, by selecting from a drop-down menu.

As desired by the workshop participants, a filter function was implemented to allow users to easily search for and filter entries. Being able to review, edit, share, or delete entries was underlined as very important, particularly from the service users’ perspective. Another important aspect highlighted by the workshop participants was the need for “hidden picture storage,” which prevents pictures from being directly transferred to the user’s phone gallery. In other words, pictures taken within the app would remain within the app and would not appear anywhere else. This hidden storage was considered to serve both to simplify documentation (all photos are in one place, making it easier to find and compare pictures) and to protect the individuals’ privacy. Several workshop participants mentioned how easily it could happen that an unpleasant and possibly embarrassing picture may pop up on the screen when showing a friend holiday pictures on one’s phone.

#### Expert Consultation

**Figure 4:**
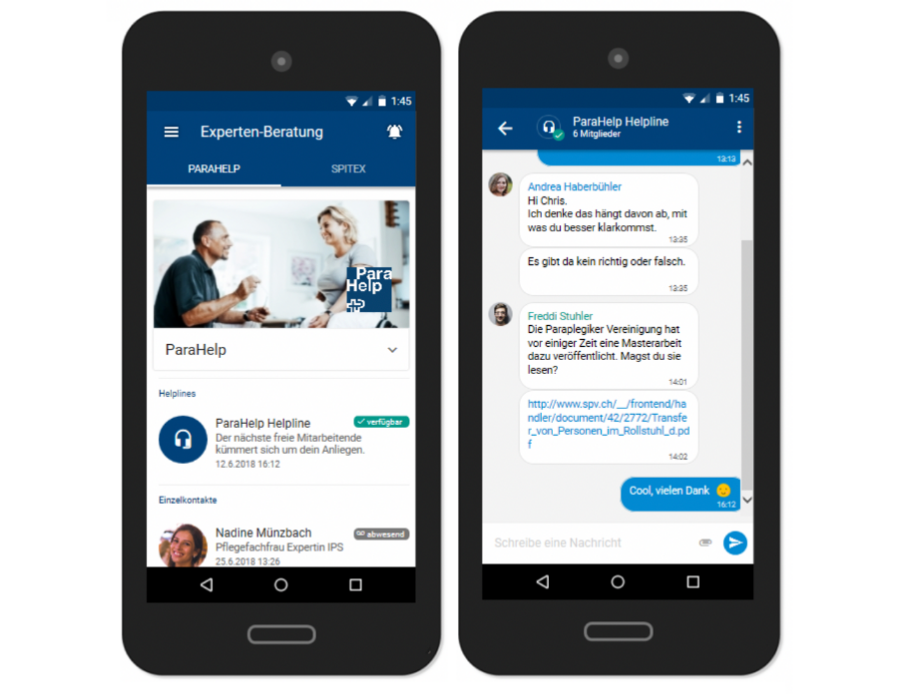
Expert consultation.

Workshop participants agreed that expert consultations constitute an integral part of the app (Figure 4). They suggested that being able to record and send audio messages would be particularly helpful for people with limited hand functioning. In terms of design and functionalities, participants referred to WhatsApp as a solution that most people are fairly familiar and comfortable with. WhatsApp was described as the current status quo medium for sharing and receiving support requests, including pictures of pressure injuries. It was thus argued that to replace this well-established practice, the app would need to provide additional benefits to users.

Individuals with spinal cord injury recognized the key benefit in the fact that all communication with health care professionals would be secure, thereby not jeopardizing their privacy. Health care professionals confirmed that a system that would allow them to display their absences would help to avoid common problems such as pictures not being received on time due to an employee’s holiday absence. A controversial aspect discussed in this context was the level of desired privacy. Although some service users argued that they would prefer to simply send an anonymous request as a way of maintaining their autonomy and decision power, health care professionals emphasized the significance of knowing who they are providing advice to. They argued that they would first need to verify whether the person contacting them is entitled to use their service, in line with their service mandate (limited to individuals with spinal cord injury). In addition, they would need access to the person’s medical history to be able to give them the best possible recommendation. However, precisely what such a registration process can and should look like was not further discussed within the scope of the workshops.

#### Reminders

**Figure 5:**
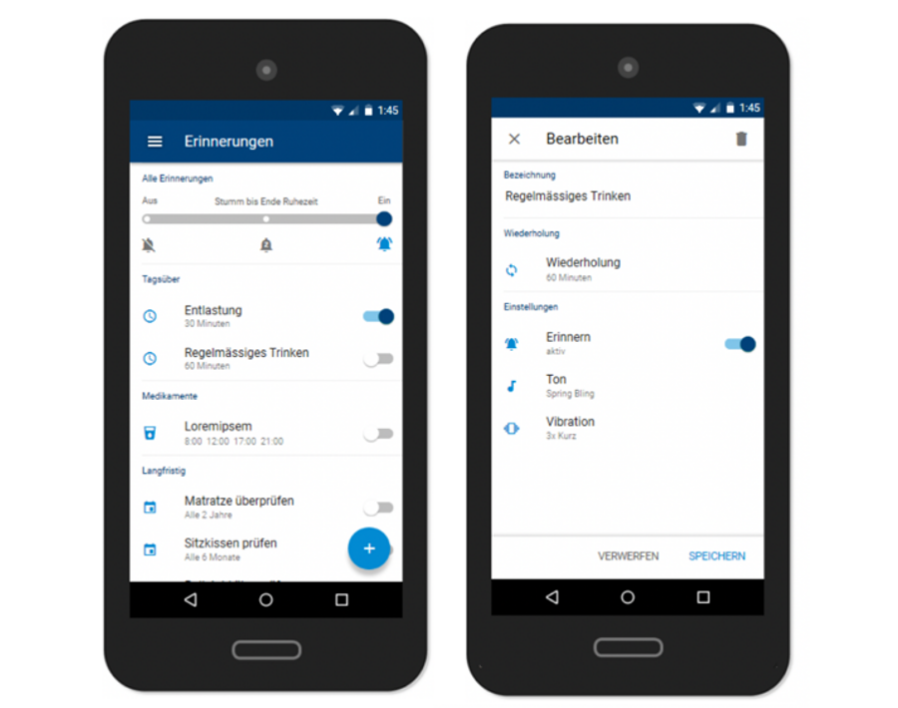
Reminders.

A somewhat more debated feature of the app was the reminder functionality (Figure 5). Workshop participants agreed that many of the guidelines for the prevention of pressure injuries could be transferred into the app in the form of reminders (eg, for regular pressure relief). Although some workshop participants identified reminders as a useful support tool, including for informal caregivers, others perceived the reminders to be patronizing and annoying, especially when presented to users in a generic form. In this sense, participants described recommendations such as “You should engage in pressure relief x times a day” as poorly tailored and likely to be ineffective. Participants agreed that, ideally, the app would include sufficient information to provide them with tailored, timely interventions (ie, provide recommendations in the moment when needed rather than at predefined times) such as “At the moment pressure on your right heel is critical, please check your skin and relieve immediately to avoid a pressure injury.” However, there was no consensus as to how this could be achieved in practice.

As a result of these discussions, the current app prototype provides users with a selection of reminder templates relating to everyday activities (eg, regular drinking, repositioning) and more long-term events (eg, check the seat cushion or mattress). Users can modify the frequency/date of each reminder. Users can also add new reminders and edit or delete them at any time. Workshop participants agreed that ideally these reminder settings should be completed together with a health care professional. In addition, the specification of resting periods during which signal tones and vibration functions are deactivated was designed to prevent users from receiving reminders unintentionally, for example during the night.

#### Knowledge Repository

In addition to specific functionalities that would translate the guidelines identified in the preparatory phase more indirectly, participants also acknowledged the importance of having a knowledge repository for finding relevant information to foster awareness of different preventive measures. It was also suggested that caregivers may benefit from being able to read up information in an easily accessible format. Workshop participants emphasized the importance of visual materials, including both images and videos. They also advised against presenting large chunks of text as users may feel overwhelmed.

**Figure 6:**
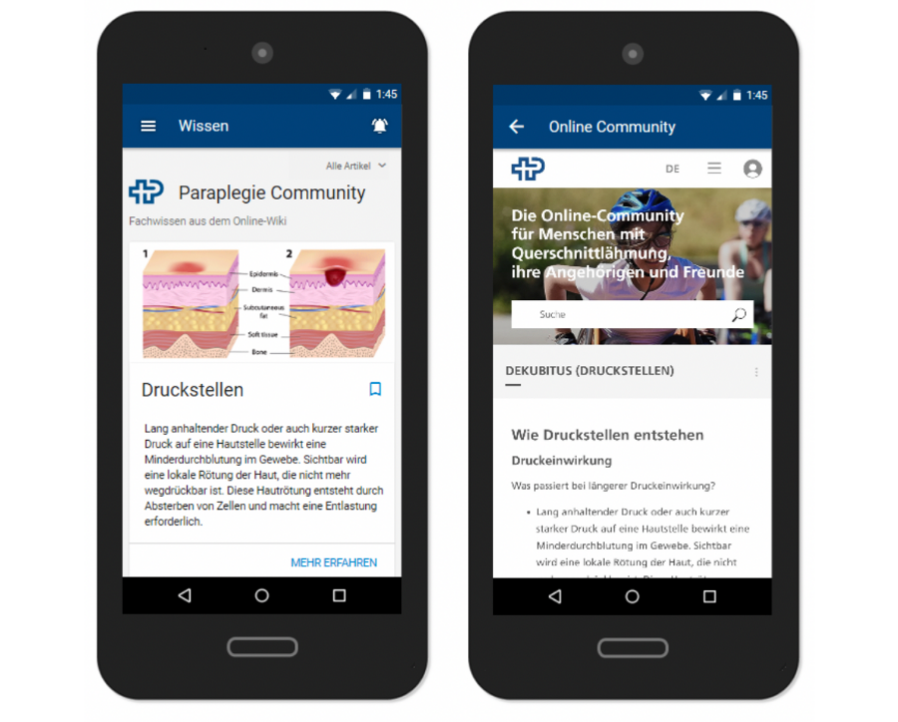
Knowledge repository.

In conceptualizing the knowledge repository (Figure 6), we drew on existing resources, namely the knowledge repository of the Paraplegie Community [32], which already provides a wide range of patient education material. The content is available in four languages (German, French, Italian, and English) and is regularly updated by experts in the field of spinal cord injury. Workshop participants agreed that it would be useful to directly incorporate the knowledge repository of the Paraplegie Community into the app prototype as it was both a fitting solution and a pragmatic choice.

### Phase 2: Perceived Usability of the App Prototype

Analysis of the task completion exercise (11 tasks) indicated a high level of usability. As shown in Table 3, most of the participants managed to complete the majority of tasks, at least partially. In cases where participants did not complete tasks, this was often caused by technical limitations of the app prototype or a misinterpretation of the task at hand. Given that participants were asked to verbalize their navigation behavior while completing the tasks (think-aloud technique), task completion times were not representative of the actual time it took participants to complete specific tasks. In some cases, participants would pause midway when performing a task to ask questions or to comment on design or navigation elements, leading to inaccurate task completion times. As a consequence, we did not analyze task completion times in-depth. Yet, our analysis of participants’ navigation recordings showed that most participants navigated the app very fluently with little back and forth navigation (Table 3). These positive results are mirrored in the average system usability score we calculated from the individual participants’ ratings displayed in Table 3. The app prototype received an average rating of 78.5/100 (range 47.5-95.0).

**Table 3:** Individual assessments of the 11 tasks.

Participant ID	Number of tasks completed	Number of tasks partially completed	Number of tasks not completed	Average navigation fluency (1-5)	SUS^a^ score (0-100)
AM0708	7	2	2	4	80
ChC1308	8	1	2	4	75
FB2908	9	2	0	4.2	87.5
FH0709	9	2	0	4.6	72.5
FR0608	9	1	1	4.4	90
GB0608	8	2	1	4.5	95
JL1409	5	4	2	4.5	75
ML2908	3	3	5	4	85
MS0908	10	0	1	4.8	80
NH0908	5	2	4	4.7	77.5
SIM1709	3	4	3	4.1	90
SM1308	6	3	2	4	75
TF0708	9	1	1	4.6	70
TH0908	6	2	3	4.4	47.5
TNS2108	7	2	2	4	77.5

^a^SUS: system usability scale.

Our analysis indicated that reoccurring challenges to the completion of tasks were primarily related to the following aspects: (1) the testing device itself (Android, as iPhone users were unfamiliar with the device, its icons, and navigation system); (2) misunderstandings or misinterpretations regarding the task (eg, participant set a reminder for a different time than requested by the task); (3) confusion caused by labeling/word choice of the app prototype (eg, the term “chat” led to confusion); (4) preconceived behaviors and preconceptions (eg, in one of the tasks, participants were asked to send a picture to a health care professional. However, instead of sending the picture through the app, some participants tried to export the picture to email); and (5) malfunctioning/limited functionality of the app prototype (eg, participant would click the “share” icon, which was not activated in the prototype). No structural differences in task completion and navigation were observed in the study population.

Findings of the task completion exercise were supported by our qualitative analysis of the semistructured interviews. Despite the limited functionality of the app prototype, most participants rated the prototype’s usability quite high. They appreciated the logical, intuitive structure and the simplicity of the app prototype, as well as the familiar functionalities.

No, super logical. Even if you come from a different corner, I go over the camera and take a new picture, or I go over to pressure injuries and take a new picture that works, that's cool.

MS0908

Well, I like it. Also, that it has some sort of chat history. That's really something most people are familiar with. These are the functions people know. It's easy. It's clear.

FB2908

Although most participants rated the usability of the app prototype as high, some of its aspects were also criticized. For example, several respondents pointed out that some content was depicted in a font size that was too small, making it difficult to read, and that it would be good to have an overview of all the things the app provides rather than having to look for it. Further criticism related to the lack of a search function to identify relevant content. Participants also noted that navigation difficulties could arise, especially for people who were not yet familiar with the app's structure.

It might be good to see the possibilities the app offers right at the beginning. To show you what you can do, otherwise, you have to click through. I think it's fine if you are familiar with it, but when you open the app for the first time you have to search for everything.

AM0708

Exactly, so it should be clearer and that you don't have to scroll too much because you lose a lot of time and you can't really read it well if you skip something.

TH0908

Similar to the findings of the usability test, our qualitative analysis of the interviews indicated that some of the navigation difficulties related to the testing device itself rather than to the navigation structure of the app prototype. One of the study participants also noted that elderly users may have difficulties navigating.

For me, the difficulty was that I don't have a Samsung. But if you have the app, it’s on your own phone and you know how [to use it]. Of course, for elderly people, it is a bit more difficult.

GB0806

Well, it was more of a fight with the Samsung [phone]. I know the iPhone by heart. If I could’ve worked with the iPhone, it would've probably been faster.

JL1409

The navigation issue was, however, described as minimally problematic by some of the participants, claiming that it was part of a natural learning process that one goes through whenever installing a new app. In this way, they suggested that users would explore the app through trial and error to get to know and understand it better.

Well, the first time you access it, it's just a little bit of trying things out, and eventually you'll get how it works.

FB2908

Then you just press something, you see what happens - what is it that I’m looking for exactly. It’s a bit like a natural flow guiding you […] It’s somehow automatic, you just try things out as you go along.

ML2908

## Discussion

### Principal Findings

Using spinal cord injury as a case in point, this study details the process and result of engaging different stakeholder groups in the development of a self-management app. Our findings illustrate how self-management needs can be translated into meaningful technical solutions by involving the relevant stakeholders in an open dialog and creative exercises. The fact that all activities were guided by the previously developed evidence-based content model (preparatory phase) helped to ensure that the app prototype would draw on the latest scientific evidence [9]. Given that many apps that address specific disability conditions are informational and provide only limited functionality, findings of this study are of great relevance to advancing the development of mHealth solutions for people with disabilities [45].

### Co-designing mHealth Solutions: Lessons Learned

In the following, we critically reflect on co-design as an approach to enrich the development of self-management apps more generally. In particular, we would like to highlight three key lessons learned from this project that are in line with recent work in the field of co-designing health services [30,46], and for specifically designing self-management support for people with spinal cord injury [28].

A first point relates to what is commonly referred to as needs assessment. Most scholars and practitioners will agree that mHealth apps should be tailored to the needs of their intended users as the end beneficiaries. For this purpose, needs assessments are carried out. Unlike other forms of needs assessments (eg, through surveys or interviews), the co-design approach we adopted in this study allowed us to understand different needs in context [31]. In other words, different stakeholders’ needs were enacted, contextualized, and put into perspective through an interactive exchange among the different stakeholders involved in the prevention and treatment of pressure injuries. Our experiences reflect those of earlier work [47], highlighting that user personas can be particularly helpful in this process as they allowed participants to think beyond their personal experiences and consider different scenarios.

A second point relates to translating needs into supporting materials. In traditional, top-down patient education, experts will—based on the needs they identified—devise educational materials to address the needs of the target population. We adopted a different approach to translating needs into support materials. More specifically, we engaged the prospective users of the app both in the identification of needs *and* in the identification of technical solutions that could help to address them. In doing so, we were able to access a previously untapped source of ideas and knowledge, resulting in a rich catalog of desirable features and functionalities of the app prototype. The user experience designer greatly facilitated the co-design process by illustrating how different technical solutions could address specific requirements or issues raised by the participants (eg, relating to data protection and privacy). As demonstrated by earlier work [48], we also observed how creativity was unleashed during the ideation workshop. Creativity-focused exercises such as drawing mock-ups of user interfaces not only stimulated discussion but also led to instances of knowledge cocreation at the intersection of lived experience, medical expertise, and technical solutions, as illustrated by the description of the individual functionalities of the app prototype.

A third point relates to our learning process as a research team. While moving along the co-design process, we as a research team were continuously confronted with our own scientifically grounded assumptions about the self-management of pressure injuries. When initially conceptualizing the app prototype, for example, we placed much greater emphasis on the self-management component of the app, neglecting the importance of communication aspects, which turned out to be an essential component. Being open to this learning process, despite being challenging at times, was indispensable and allowed us to collaborate with our study participants on eye level. During the co-design process, it also became evident that there are conflicting concepts and desires, not only between the different stakeholder groups but also within the same groups. The prime example to mention here is that people with spinal cord injury requested timely interventions (rather than arbitrary, generic advice), which is difficult if not impossible to reconcile with their wish for as much autonomy and privacy as possible. In addition, the conflicting nature of some evidence-based guidelines was identified as a key challenge [9]. In trying to manage these tensions and possible points of conflict, we also experienced the dark sides of co-design [29,49]. We had to accept and deal with the fact that consensus among all stakeholders was not always attainable nor compatible with the financial and organizational constraints we faced [50,51]. This involved negotiation, mediation, and, most importantly, managing participants’ and other stakeholders’, including funders’, expectations [52]. In line with earlier work [53], we also had to face the fact that our co-design and development approach had failed to adequately consider the need for an implementation plan and a business model.

Our values, knowledge, and experiences as researchers inevitably shape our work in one way or another [54,55]. Verbalizing our assumptions and expectations regarding the co-design process and its outcomes from the very beginning can help us to establish a baseline. Against this baseline, we can then consider the added value of co-design by asking ourselves: What specifically did we gain from involving different stakeholders at eye level? What are the aspects we would have likely failed to consider? Could some of our taken-for-granted assumptions be refuted? Moreover, being conscious and reflective about the underpinnings of our work can help us to maintain our research integrity and minimize the risk of tokenism when co-designing health solutions with different stakeholders. Critical reflection can also promote out-of-the-box thinking, a much-needed skill when addressing design problems that could be tackled by a myriad of potential solutions [56,57]. Without such critical reflection, research teams may fail to explore alternative solutions and instead fixate on preset notions and ideas, which may result in suboptimal design and development choices [58]. To promote such out-of-the-box thinking, we chose to prioritize stakeholders’ lived experience and clinical guidelines instead of relying on existing theoretical models to inform the design and development of the app prototype. At a next stage, we plan to link our findings to existing behavior change theories [59] to devise detailed intervention content for a pilot trial. This will then allow us to perform a rigorous evaluation of our self-management app and help us to identify its most effective components [60].

### Strengths and Limitations

It needs to be acknowledged that the individuals agreeing to take part in this study may have been more technology savvy and may have thus held more positive attitudes toward mHealth solutions as compared to those individuals that did not take part. As a consequence, the app prototype may fail to account for the specific needs of less tech-savvy target audiences. To counteract this limitation, we incorporated user personas in the co-design process to allow and encourage participants to think beyond their personal expectations and experiences with technology. Moreover, there are also some limitations related to the functionality of the app prototype used to carry out the usability tests. Given that the app prototype was web-based, functionality was limited and sometimes impeded, which may have led to poorer evaluations. To account for this, all participants were made aware of these inherent limitations of the technology before starting the usability test. From a methodological point of view, combining the task completion exercise with the think-aloud technique during usability testing presented some limitations. More specifically, it prevented us from assessing the actual time it would take a participant to complete a specific task. Last but not least, we cannot be sure how truly participatory our co-design approach was. Although we aimed to involve the different stakeholder groups as equal partners, we cannot know with certainty whether this is how they experienced the co-design process. We thus recommend researchers using co-design methodologies to incorporate process evaluations into their research to gain a better understanding of how different stakeholders experience their participation.

### Conclusions

Spinal cord injury is a complex chronic health condition, requiring those affected to navigate competing interests and priorities, paired with uncertainty about the accuracy and relevance of clinical recommendations. This study shows that involving individuals with spinal cord injury and health care professionals in co-designing a self-management app is both a feasible and enriching exercise in that it fosters knowledge cocreation at the intersection of lived experience, medical expertise, and technical solutions. In light of a current dearth of mHealth solutions tailored to the needs of community-dwelling individuals with spinal cord injury, this study makes an important contribution by advancing our knowledge on how to design interventions that can motivate behavior change, specifically regarding the prevention of pressure injuries. However, co-designing self-management solutions is a time and resource-intensive endeavor. Future research is needed to evaluate the impact of a co-designed self-management app and to demonstrate its additional value over conventional top down–designed solutions.

## Appendix

Multimedia Appendix 1 Ideation Workshop.

Multimedia Appendix 2 Usability Test.

Multimedia Appendix 3 Interview Guide.

Multimedia Appendix 4 Core Themes and matching Functions.

## Abbreviations

mHealth: mobile health
