# Supplementary material for: Co-designing a Self-Management App Prototype to Support People With Spinal Cord Injury in the Prevention of Pressure Injuries: Mixed Methods Study
Source: JMIR Mhealth Uhealth. 2020 Jul 9;8(7):e18018. doi: 10.2196/18018 (PMC7380902; doi:10.2196/18018)

Multimedia Appendix 1

Ideation Workshop

Prevention types and their (assumed) preferences

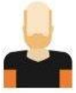

Delegierende

Trifft hauptsächlich "passive" Präventionsmassnahmen und delegiert an unterstützende Angehörige und Spitex z. B. er hat eine Luftmatratze gekauft und ist wütend wenn trotz der Matratze ein Dekubitus entsteht.

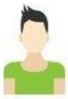

Selektive

Trifft einige der empfohlenen Präventionsmassnahmen, basierend auf seinem Erfahrungswissen z. B. er kontrolliert nicht regelmässig die Stellen, wo er nie etwas gehabt hat.

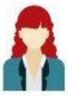

Umsichtige

Trifft alle empfohlenen Präventionsmassnahmen.

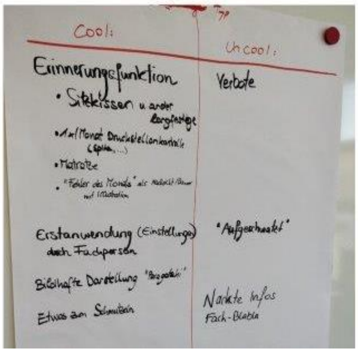

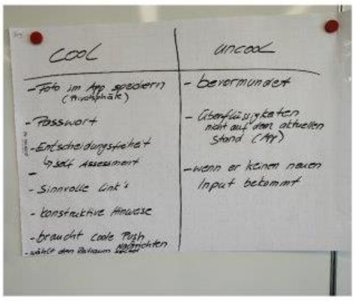

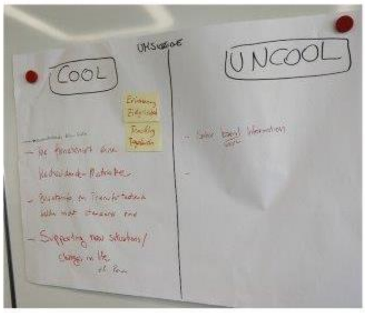

Generating ideas for "translation" of clinical guidelines into app content and functions

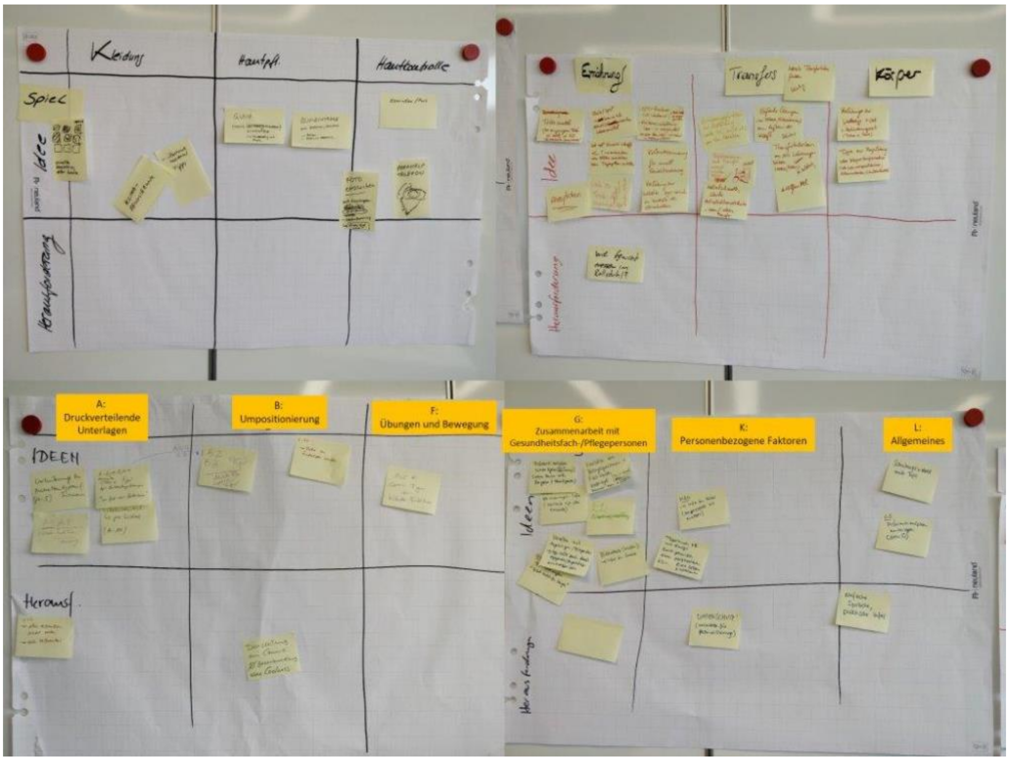

Love Letter

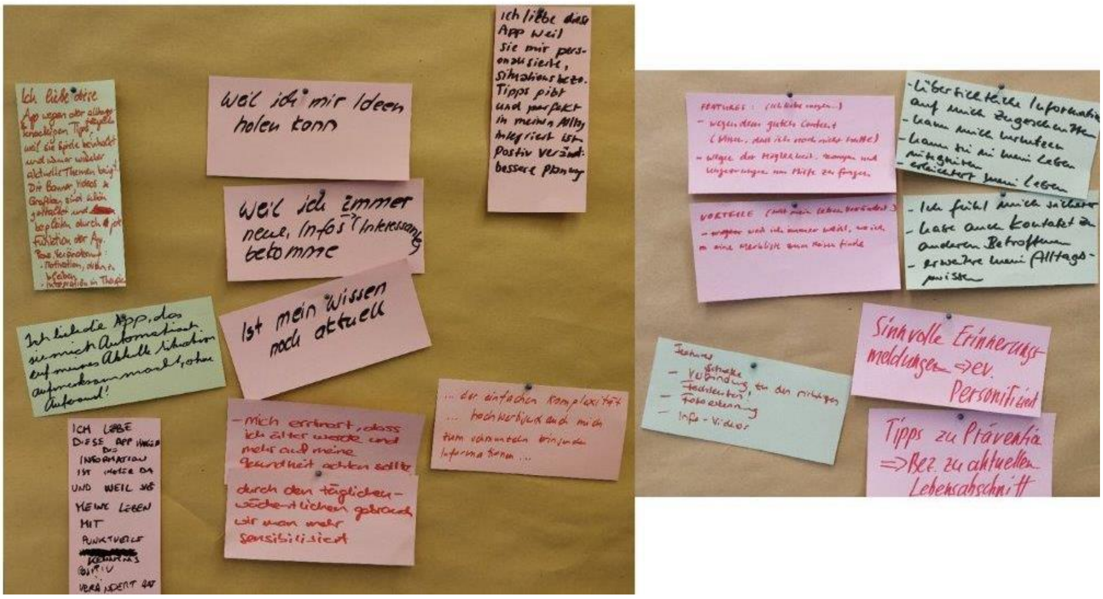

"One-star-review"

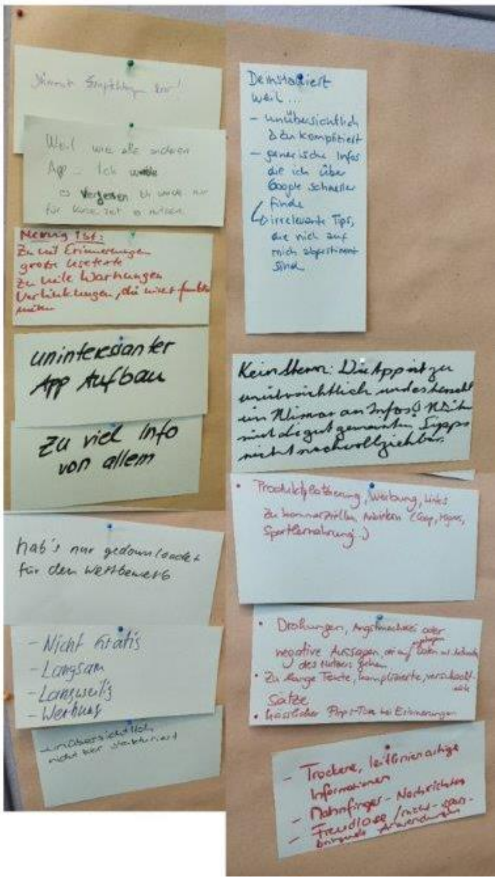

User-interface sketching

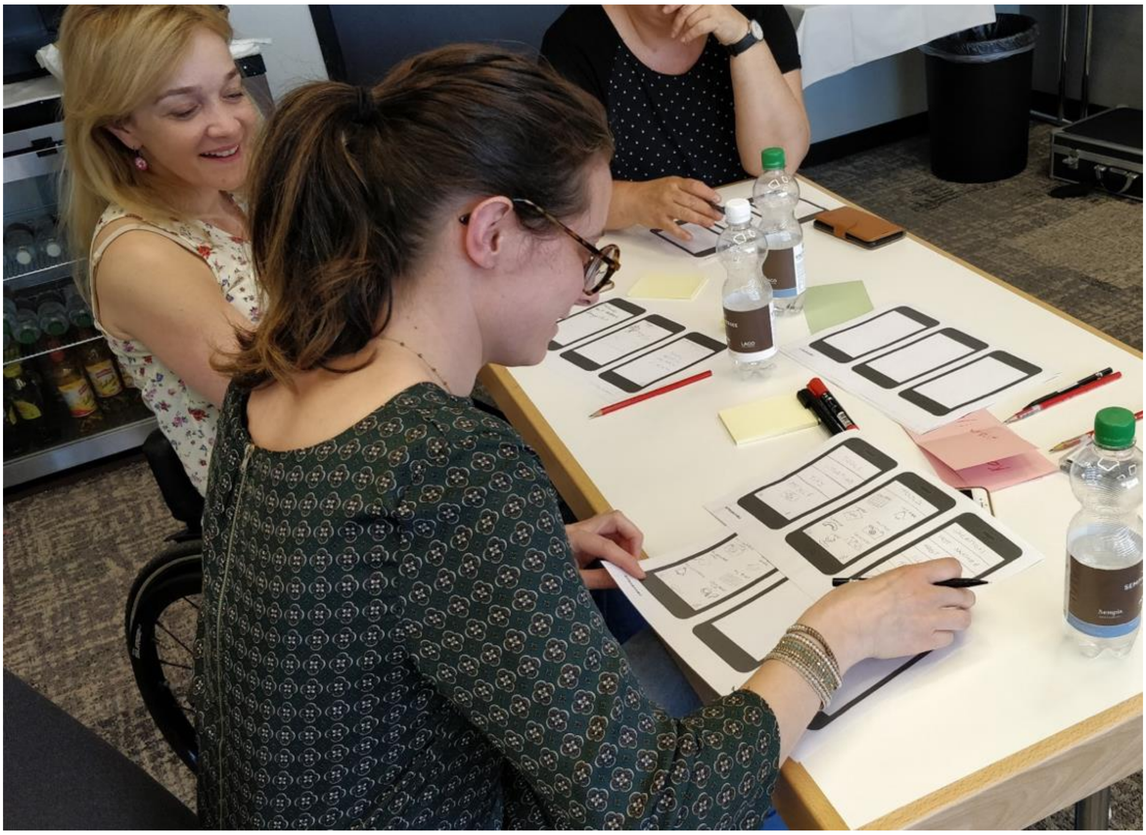

Supplement: Multimedia Appendix 1 [file mhealth_v8i7e18018_app1.pdf]
