# Supplementary figures and images for: Co-designing a Self-Management App Prototype to Support People With Spinal Cord Injury in the Prevention of Pressure Injuries: Mixed Methods Study
Source: JMIR Mhealth Uhealth. 2020 Jul 9;8(7):e18018. doi: 10.2196/18018 (PMC7380902; doi:10.2196/18018)

# Multimedia Appendix 2

## Usability Testing

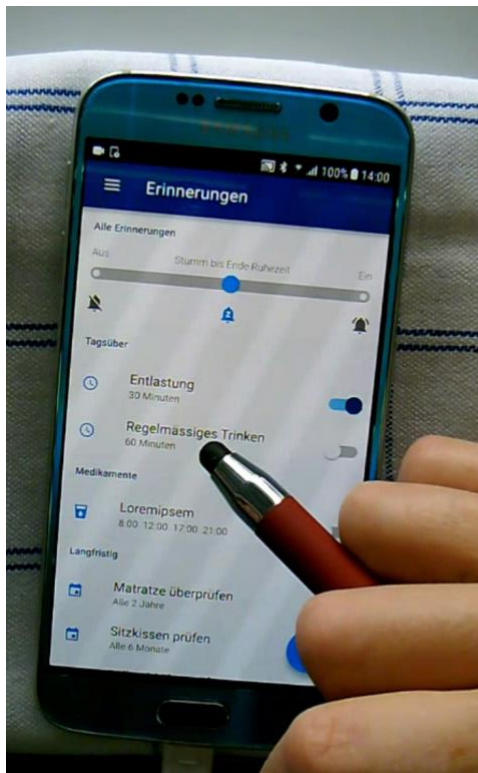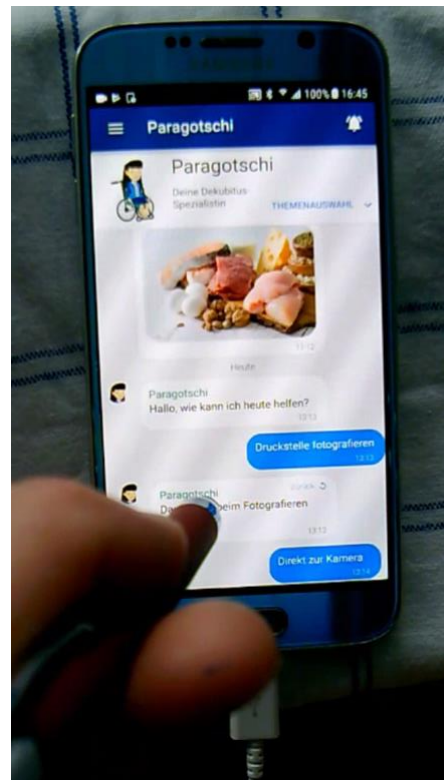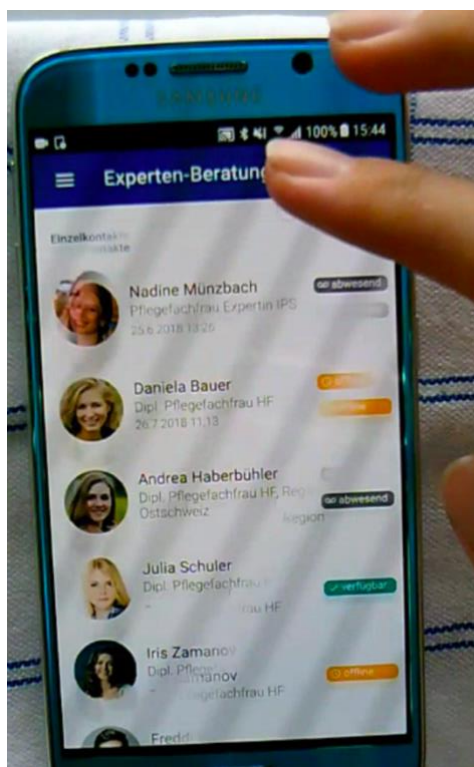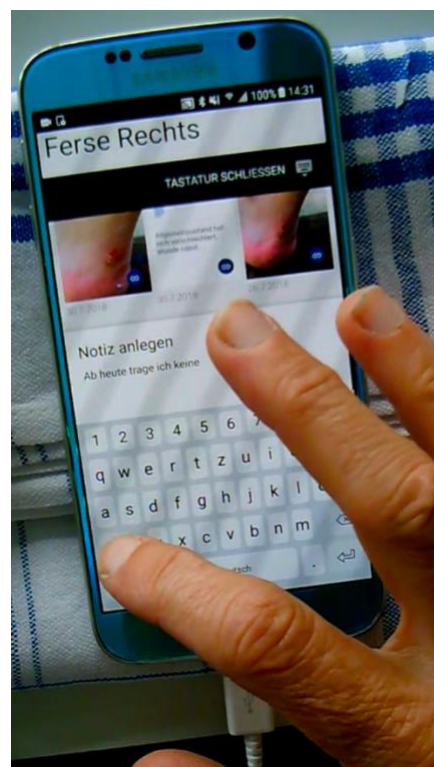

Supplement: Multimedia Appendix 2 [file mhealth_v8i7e18018_app2.pdf]
