# Supplementary material for: Co-designing a Self-Management App Prototype to Support People With Spinal Cord Injury in the Prevention of Pressure Injuries: Mixed Methods Study
Source: JMIR Mhealth Uhealth. 2020 Jul 9;8(7):e18018. doi: 10.2196/18018 (PMC7380902; doi:10.2196/18018)
Supplement: Multimedia Appendix 3 [file mhealth_v8i7e18018_app3.pdf]

Multimedia Appendix 3

Interview guide

1. **Experience in using apps and specifically health apps**
   a) Do you use apps in your everyday life? If so, what kind of apps?
   b) Do you also use health apps? If so, which ones (and for what)?
   c) Why do you use (no) health apps? What kind of experience have you had?

2. **General assessment of prototype**
   a) What is your first impression of the app prototype?

3. **Perceived usability of the app prototype**
   a) Did you have difficulties in completing the 11 tasks?
   b) How did you feel about handling and navigation of the app?
   c) Did you find your way around the app well or not so well?
   d) Did you find the app clear or rather confusing?
   e) Were there things that surprised you or that you did not understand?

4. **Perceived benefits of the app**
   a) How would you use the app in your everyday life?
   b) Are there certain features or content that you find particularly useful, and if so, why?
   c) Are there any situations where the app could be particularly useful in your daily life?
   d) Would you recommend the app to someone? If so, to whom and why?

5. **Attitudes towards using the app**
   a) Where do you see the advantages and disadvantages of the app?
   b) Do you see any risks?

6. **Intention to use the app**
   a) Can you imagine using the app in your everyday life in the future?
   b) If not, why not?
   c) Would you use this app to communicate with healthcare professionals?
      i. If so, what do you see as the benefits?
      ii. If not, why not? What risks do you see?

7. **Suggestions for improvement**
   a) What did you particularly like?
   b) Was something missing?
