# Supplementary material for: Co-designing a Self-Management App Prototype to Support People With Spinal Cord Injury in the Prevention of Pressure Injuries: Mixed Methods Study
Source: JMIR Mhealth Uhealth. 2020 Jul 9;8(7):e18018. doi: 10.2196/18018 (PMC7380902; doi:10.2196/18018)
Supplement: Multimedia Appendix 4 [file mhealth_v8i7e18018_app4.pdf]

Multimedia Appendix 4

Core Themes and matching Functions (Phase 1)

**Prospective users' needs/desires**	**App prototype functions**	
Self-management component (individual)		
Assist disease monitoring	Smart camera	Pressure injury diary
Provide disease knowledge	Knowledge repository	Reminders
Provide motivational support	Reminders	
Communication component (interaction)		
General expert inquiries	Expert consultation	
Acute expert feedback	Expert consultation	
